# The effect of sham acupuncture can differ depending on the points needled in knee osteoarthritis: A systematic review and network meta-analysis

**DOI:** 10.1016/j.heliyon.2024.e25650

**Published:** 2024-02-07

**Authors:** Boram Lee, Chan-Young Kwon, Hye Won Lee, Arya Nielsen, L Susan Wieland, Tae-Hun Kim, Stephen Birch, Terje Alraek, Myeong Soo Lee

**Affiliations:** a KM Science Research Division, Korea Institute of Oriental Medicine, Daejeon, Republic of Korea; b Department of Oriental Neuropsychiatry, Dong-eui University College of Korean Medicine, Busan, Republic of Korea; c KM Convergence Research Division, Korea Institute of Oriental Medicine, Daejeon, Republic of Korea; d Department of Family Medicine & Community Health, Icahn School of Medicine at Mount Sinai, New York, USA; e Center for Integrative Medicine, University of Maryland School of Medicine, Baltimore, MD, USA; f Korean Medicine Clinical Trial Center, Korean Medicine Hospital, Kyung Hee University, Seoul, Republic of Korea; g Kristiania University College, School of Health Sciences, Oslo, Norway; h Department of Community Medicine, Faculty of Health Sciences, National Research Center in Complementary and Alternative Medicine (NAFKAM), Institute of Health Sciences, Tromsø, Norway

**Keywords:** Acupuncture therapy, Knee osteoarthritis, Placebo, Sham acupuncture, Network meta-analysis

## Abstract

**Objective:**

In sham acupuncture-controlled acupuncture clinical trials, although sham acupuncture techniques are different from those of verum acupuncture, the same acupuncture points are often used for verum and sham acupuncture, raising the question of whether sham acupuncture is an appropriate placebo. We aimed to examine the effects of sham and verum acupuncture according to the points needled (same or different between verum and sham acupuncture) in knee osteoarthritis.

**Methods:**

Ten databases were searched to find randomized controlled clinical trials (RCTs) assessing the effects of verum acupuncture with sham acupuncture or waiting lists on knee osteoarthritis. Sham acupuncture was classified as using the same acupuncture points as those in verum acupuncture (SATV) or using sham points (SATS). A frequentist network meta-analysis (NMA) was conducted, and the certainty of evidence was evaluated.

**Results:**

A total of 10 RCTs involving 1628 participants were included. Verum acupuncture was significantly superior to SATS but not different from SATV in terms of pain reduction. Additionally, SATV was significantly superior to the waiting list. For physical function, no difference were found between verum acupuncture, SATV, and SATS. The certainty of evidence was low to moderate.

**Conclusion:**

For knee osteoarthritis, the pain reduction effect of acupuncture may differ according to the needling points of sham acupuncture, and the control group should be established according to the specific aim of the study design and treatment mechanism.

## Highlights

• Some trials used the same acupuncture points for both sham and verum acupuncture groups.
• Sham acupuncture may have different outcomes by points needled on knee osteoarthritis.
• Any stimulation of the same points as the verum acupuncture arm may not be a true placebo.

## 1 Introduction

Randomized placebo-controlled trials are considered the gold standard for evaluating the efficacy of a particular intervention. The use of a placebo control ensures that nonspecific effects (e.g., expectation) are similar between treatment groups and allows the isolation of the specific effects of an active treatment. Acupuncture therapy, involving needle insertion at strategic points on the body, has been used for a long time for the treatment and management of various diseases, and many trials have been conducted to evaluate its efficacy. However, as multiple factors influence the effect of therapies including acupuncture, such as number and specificity of acupuncture points, needle skin penetration, needle manipulation, time of needle retention, and patient-therapist psychological interaction [1,2], questions have been raised as to whether an appropriate “placebo acupuncture” that can control for all of these factors is possible [3].

In randomized controlled clinical trials (RCTs) for evaluating the efficacy of acupuncture, sham acupuncture has been used as a control intervention. Non-penetrating sham acupuncture devices and shallow needling have been used as variants of sham acupuncture, each applying the techniques of acupuncture differently from those of verum acupuncture; however, they have not been established as physiologically inert placebos [4]. Furthermore, some studies used the same acupuncture points for both sham acupuncture and verum acupuncture groups [4]. In such a case, among the various factors known to induce the effects of acupuncture, the acupuncture points were not controlled; thus, sham acupuncture cannot be considered a placebo control [3,4]. We assumed that there would be a difference in effects depending on the points needled in sham acupuncture (same acupuncture points as those in verum acupuncture or other points not indicated to have a therapeutic effect on the disease or condition). To investigate this, a network meta-analysis (NMA) was conducted for chronic nonspecific low back pain (cLBP) in a previous study [5]. In this study, the effects of verum acupuncture in improving pain and function in cLBP were different depending on the points needled in sham acupuncture, and there was a difference in effects between the two types of sham acupuncture. Therefore, we aimed to investigate whether the same results would be obtained for knee osteoarthritis, another chronic pain condition for which acupuncture has been widely used with inconsistent recommendations across clinical practice guidelines due to the inconsistent effects between acupuncture and sham acupuncture in different RCTs [[6], [7], [8], [9]]. As far as we know, no direct comparative RCT has been conducted according to the points needled in sham acupuncture for knee osteoarthritis; thus, we examined the effects through a NMA, which allows indirect and mixed comparisons.

## 2 Methods

The protocol was registered with PROSPERO (registration number: CRD42023405497).

### 2.1 Inclusion and exclusion criteria

Prospective RCTs involving adult patients with knee osteoarthritis without limitation on sex, age, and nationality were eligible. The intervention and comparator included verum acupuncture, sham acupuncture, and waiting list. Verum acupuncture consisted of only manual acupuncture with inserted needles. According to our research hypothesis, sham acupuncture was classified into the following two types based on the points needled: (1) SATV: sham acupuncture needling at the same verum acupuncture points as those in the verum acupuncture; (2) SATS: sham acupuncture needling at sham points, different from those in the verum acupuncture. In addition, to form a connected loop on the network plot and to compare the real-world effectiveness of the verum and sham acupuncture groups, the waiting list group was included as intervention and comparator.

Post-intervention pain intensity was the primary outcome, measured by such as the Western Ontario and McMaster Universities Osteoarthritis Index (WOMAC) pain subscale, Numerical Rating Scale (NRS), Brief Pain Inventory, and Visual Analog Scale (VAS). Post-intervention physical function was the secondary outcome, measured by such as the WOMAC function subscale. When our outcomes of interest were assessed with multiple outcome measures, we prioritized the WOMAC scale if it was available. We chose to analyze the first results after completion of all treatment sessions.

### 2.2 Information source and database search

The following 10 electronic databases were searched on January 28, 2023: Medline, Cochrane Central Register of Controlled Trials (CENTRAL), EMBASE, Allied and Complementary Medicine Database (AMED), Oriental Medicine Advanced Searching Integrated System (OASIS), Koreanstudies Information Service System (KISS), Korean Medical Database (KMbase), ScienceON, China National Knowledge Infrastructure (CNKI), and CiNii. The detailed search strategies and the search results in all databases are described in Supplement 1. Additionally, the reference lists of related studies and trial registries were searched to identify additional studies. We included all relevant literature, including gray literature, without restriction on publication language or publication status.

### 2.3 Study selection and data collection

Study selection and data extraction process were independently performed by two researchers (BL and CYK). Any disagreements between them were reached through discussion with the other authors. All references identified through database search were imported into EndNote 20 (Clarivate Analytics, Philadelphia, PA, USA), and potential eligibility was confirmed through a review of titles and abstracts. Subsequently, the full texts were retrieved for eligible studies, and through this, the final included literature was determined. The following data were extracted for the final included literature: first author, year of publication, country in which the study was conducted, sample size, mean age of population, study comparison, details of verum acupuncture and sham acupuncture, outcomes of interest, and results.

### 2.4 Quality assessment

The methodological quality was assessed for the studies included based on the Cochrane Risk of Bias 2 tool [10]. Each five domains were assessed as “high risk of bias”, “some concerns”, or “low risk of bias”. Especially, for the domain “bias due to deviations from intended interventions”, we focused on quantifying the effect of assignment to the interventions at baseline. Considering the judgments of each domain, the overall risk of bias was determined.

### 2.5 Data analysis

For direct comparisons using the same types of interventions and outcomes, pairwise meta-analysis was performed with Review Manager 5.4 (Cochrane, London, UK). To calculate indirect and mixed estimates, a frequentist NMA was conducted using the network packages in Stata/MP 16.1 (StataCorp LLC, College Station, TX, USA) after testing for similarity, transitivity, and consistency. In particular, consistency was statistically tested using the design-by-treatment interaction model (global approach) and the node-splitting method (local approach), and a NMA was performed only when assumptions were satisfied with both approaches. The number of included studies and participants for each intervention in the NMA was represented by a four-node network map (verum acupuncture vs. SATV vs. SATS vs. waiting list). A random-effects model was selected for both pairwise meta-analysis and NMA considering unavoidable clinical heterogeneity among studies. The analysis results for continuous variables are presented using the standardized mean difference (SMD) and 95% confidence interval (CI) considering that different evaluation tools were used between studies. The effect estimates of meta-analysis are presented through league tables and interval plots on each outcome of interest. Potential publication bias was tested by funnel plot symmetry and Egger’s test if sufficient studies (n ≥ 10) were included in an analysis. The surface under the cumulative ranking curve (SUCRA) was determined for each outcome to identify the optimal treatment, and clustered ranking for the pain and physical function outcomes based on cluster analysis of SUCRA values was presented as a clustered ranking plot. Tests were 2-sided, and a *P* value < .05 was considered significant.

### 2.6 Certainty of evidence

The certainty of evidence of NMA findings was assessed according to the Grading of Recommendations Assessment, Development, and Evaluation (GRADE) method [11,12]. First, in each comparison of individual outcomes of interest, the risk of bias, indirectness, inconsistency, and publication bias of direct estimates were evaluated. In addition, the certainty of indirect estimates was determined considering intransitivity and the lowest of the ratings of the two direct comparisons forming the most dominant first-order loop. Finally, the certainty of evidence for network estimates was rated by considering the highest rating between indirect and direct ratings, imprecision of NMA results, and incoherence. The certainty of evidence for each estimate was presented as very low, low, moderate, or high.

## 3 Results

### 3.1 Study selection

**Fig. 1:**
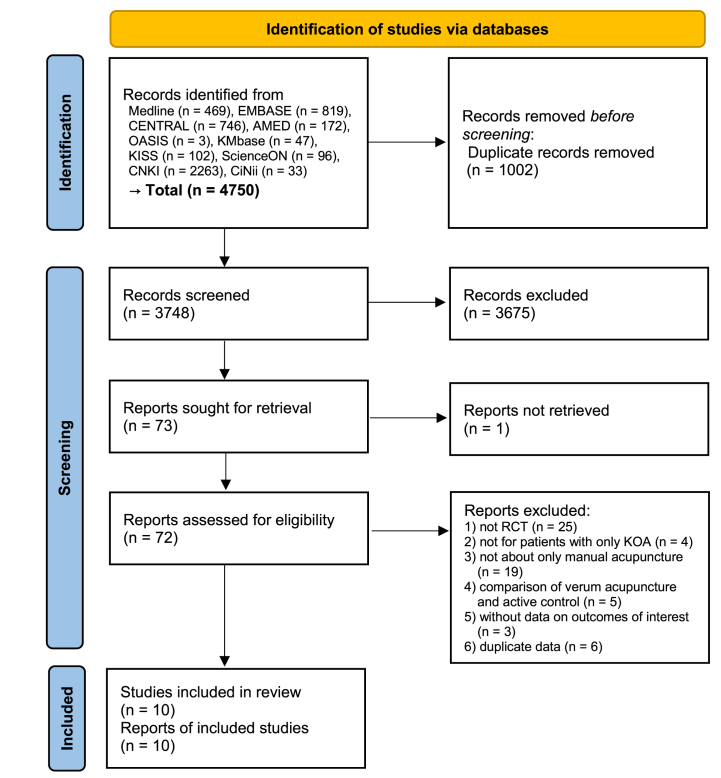
Flow diagram of the literature screening and selection processes.

A total of 4750 records were retrieved from the database search, and no studies were identified from other sources. A total of 1002 records were excluded using EndNote's “Find duplicate” function. The titles and abstracts of the remaining 3748 studies were reviewed, and 3675 studies not relevant to topic were excluded. Among the remaining 73 studies, except for 1 study whose full text was not retrieved, the full texts of the remaining 72 studies were reviewed. As a result, 62 studies were excluded: 25 non-RCTs, 4 studies not about patients with only knee osteoarthritis, 19 studies not about manual acupuncture only, 5 studies comparing verum acupuncture and active controls, 3 studies without data on outcomes of interest, and 6 studies with duplicate data (Supplement 2). Finally, a total of 10 studies [[13], [14], [15], [16], [17], [18], [19], [20], [21], [22]] comprising 1628 participants were included (Fig. 1).

### 3.2 Study characteristics

A total of 3 studies were conducted in China [14,15,18]. In addition, 2 studies were conducted in Germany [20,21], and 1 study was conducted each in Australia [13], South Korea [16], Canada [17], England [19], and Taiwan [22]. A total of 9 studies were 2-arm RCTs, among which 3 studies compared the verum acupuncture and waiting list groups [13,19,21], and 6 studies compared the verum and sham acupuncture groups [[14], [15], [16], [17], [18],^22^]. There was a 3-arm RCT comparing the verum acupuncture, sham acupuncture, and waiting list groups [20]. In terms of the technique of sham acupuncture, shallow needling was performed in 4 studies [15,17,18,20], non-penetrating needles were used in 1 study [14], and the Park sham acupuncture device (a non-penetrating needle with a base device creating a sensation that the participant is unable to distinguish it from penetration) was used in 1 study [16]. One study used vague expression of inserting the needles superficially without penetrating the skin, as the technique of sham acupuncture [22]. In terms of the points needled for sham acupuncture, 2 studies [14,16] performed SATV, and 5 studies [15,17,18,20,22] performed SATS. The basic characteristics and details of verum acupuncture methods of the included studies are presented in Table 1 and Supplement 3.

**Table 1:** Characteristics of included studies.

Study ID (Country)	Sample size (analyzed)	Mean age (yr)	(A) AT group	(S) Sham AT group		(W) Waiting list group	Treatment duration	Outcomes of interest	Time point included in the analysis
				Style	Protocol				
Hinman 2014 (Australia)	(A) 64, (W) 69	(A) 64.3 ± 8.6 (W) 62.7 ± 8.7	AT	–	–	Waiting list	12 weeks	WOMAC (pain, physical function), 0–10 NRS (pain)	12 weeks
Lam 2021 (China)	(A) 42, (S) 41	(A) 62.7 ± 7.0 (S) 63.4 ± 6.7	AT	SATV	Non-penetrating needle at the same acupuncture points as the AT group	–	4 weeks	0–100 mm VAS (pain)	4 weeks
Lin 2018 (China)	(A) 21, (S) 21	(A) 59.5 ± 7.5 (S) 60.0 ± 7.3	AT	SATS	Shallow needling at non-acupuncture points	–	8 weeks	WOMAC (pain, physical function), 0–100 mm VAS (pain)	8 weeks
Min 2006 (South Korea)	(A) 40, (S) 38	(A) 58.9 ± 5.6 (S) 60.0 ± 5.0	AT	SATV	Non-penetrating Park's sham needle at the same acupuncture points as the AT group	–	4 weeks	WOMAC (pain, physical function), 0–100 mm VAS (pain)	4 weeks
Takeda 1994 (Canada)	(A) 20, (S) 20	(A) 63.00 ± 8.78 (S) 60.20 ± 9.75	AT	SATS	Superficial needling at non-acupuncture points	–	3 weeks	WOMAC (pain, physical function)	3 weeks
Tu 2021 (China)	(A) 145, (S) 146	(A) 63.0 ± 7.2 (S) 62.8 ± 7.6	AT	SATS	Superficial needling at non-acupuncture points	–	8 weeks	WOMAC (pain, physical function), 0–10 NRS (pain)	8 weeks
Williamson 2007 (England)	(A) 60, (W) 61	(A) 72.4 ± 7.71 (W) 69.6 ± 10	AT	–	–	Waiting list	6 weeks	0–10 cm VAS (pain)	7 weeks
Witt 2005 (Germany)	(A) 145, (S) 73, (W) 67	(A) 64.5 ± 6.4 (S) 63.4 ± 6.6 (W) 63.6 ± 6.7	AT	SATS	Superficial needling at non-acupuncture points	Waiting list	8 weeks	WOMAC (pain, physical function)	8 weeks
Witt 2006 (Germany)	(A) 235, (W) 228	Not reported	AT	–	–	Waiting list	3 months	WOMAC (pain, physical function)	3 months
Yu 2021 (Taiwan)	(A) 61, (S) 31	(A) 64.79 ± 9.86 (S) 66.35 ± 10.56	AT	SATS	Non-penetrating needles at different acupuncture points as the AT group (CV12, ST21)	–	1 day	0–10 cm VAS (pain)	1 day

AT, acupuncture therapy; NRS, numeric rating scale; SATS, sham acupuncture needling at points different from those in the verum acupuncture group; SATV, sham acupuncture needling at the same acupuncture points as those in the verum acupuncture group; VAS, visual analog scale; WOMAC, Western Ontario and McMaster Universities Arthritis Index.

**Fig. 2:**
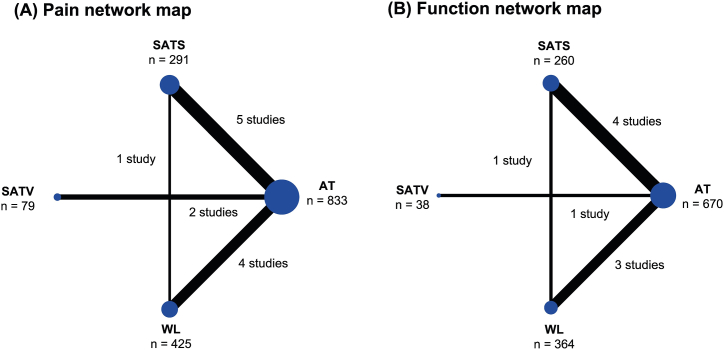
Network map of (A) pain and (B) physical function. AT, acupuncture therapy; SATS, sham acupuncture needling at points different from those in the verum acupuncture group; SATV, sham acupuncture needling at the same acupuncture points as those in the verum acupuncture group; WL, waiting list.

Pain intensity was evaluated in all studies, which included the WOMAC pain subscale in 7 studies [13,[15], [16], [17], [18],^20^,^21^], the VAS in 5 studies [[14], [15], [16],^19^,^22^], and the NRS in 2 studies [13,18]. A 4-node network map comprising verum acupuncture, SATS, SATV, and waiting list was constructed for pain outcome (Fig. 2(A)). There was no inconsistency in both the global approach (*P* value = .1153) and local approach according to the node-splitting method (Supplement 4). A total of 7 studies [13,[15], [16], [17], [18],^20^,^21^] evaluated physical function using the WOMAC subscale, of which 4 studies used the WOMAC subscale with a 0 to 4 Likert scale [13,15,16,18], 1 study used a 100 mm VAS [17], and 2 studies had no relevant information [20,21]. A 4-node network map was also constructed for physical function outcome (Fig. 2(B)). There was no inconsistency in either the global approach (*P* value = .1776) or local approach (Supplement 4).

### 3.3 Quality assessment

**Fig. 3:**
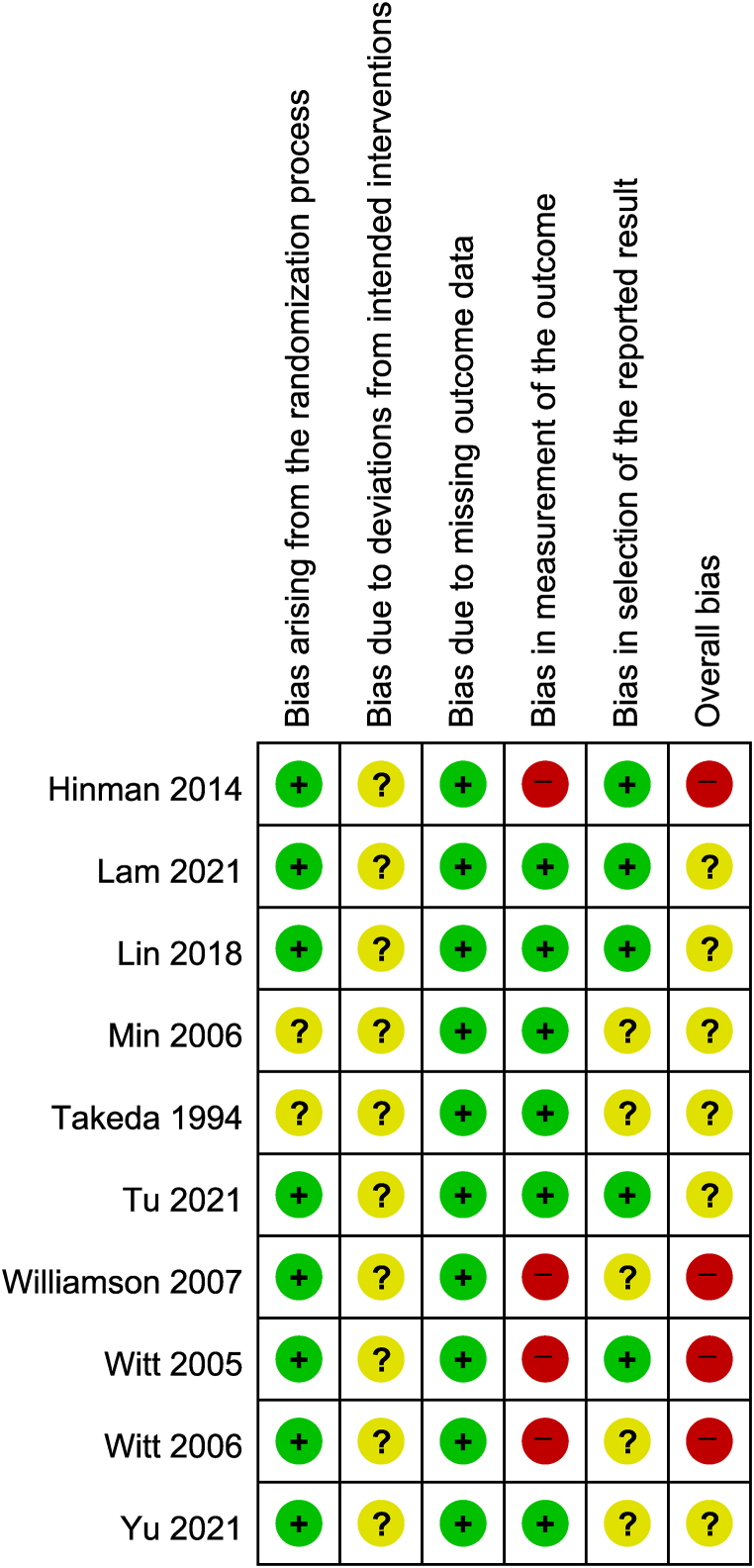
Risk of bias summary for all included studies. Low risk of bias, some concerns, and high risk of bias, respectively, are represented with the following symbols: “+”, “?”, and “-”.

No studies showed differences in baseline clinical characteristics between the groups. However, 2 studies [16,17] lacked information on allocation concealment and were assessed as having some concerns in bias arising from the randomization process. Intervention providers (acupuncturists) were aware of the intervention assigned to participants in all studies because provider blinding was not possible in acupuncture trials, and there was no information about deviations that occurred in the clinical trial context. Therefore, all studies were judged to have some concerns of risk of bias due to deviations from intended interventions. Because 4 studies [13,[19], [20], [21]] included waiting list groups, it was not possible to blind the participants. Since the outcome of interest in this study is a patient-reported scale, they were judged as having a high risk of bias in the measurement of the outcome [13,[19], [20], [21]]. In 5 studies [16,17,19,21,22] without information about prior planning for data analysis, there were some concerns of bias in selection of the reported result. Six studies [[14], [15], [16], [17], [18],^22^] had some concerns of overall bias, and 4 studies [13,[19], [20], [21]] were judged to have a high risk of overall bias (Fig. 3).

### 3.4 Data analysis: pain outcome

**Fig. 4:**
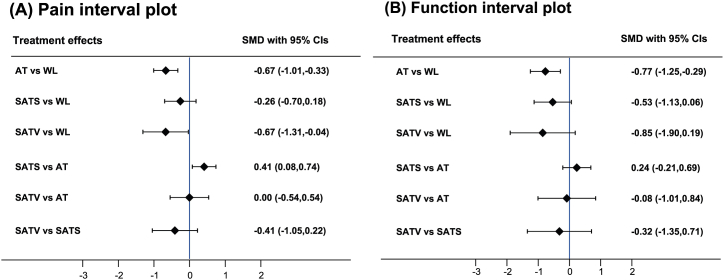
Interval plots of (A) pain and (B) physical function. AT, acupuncture therapy; CI, confidence interval; SATS, sham acupuncture needling at points different from those in the verum acupuncture group; SATV, sham acupuncture needling at the same acupuncture points as those in the verum acupuncture group; SMD, standardized mean difference; WL, waiting list.

**Fig. 5:**
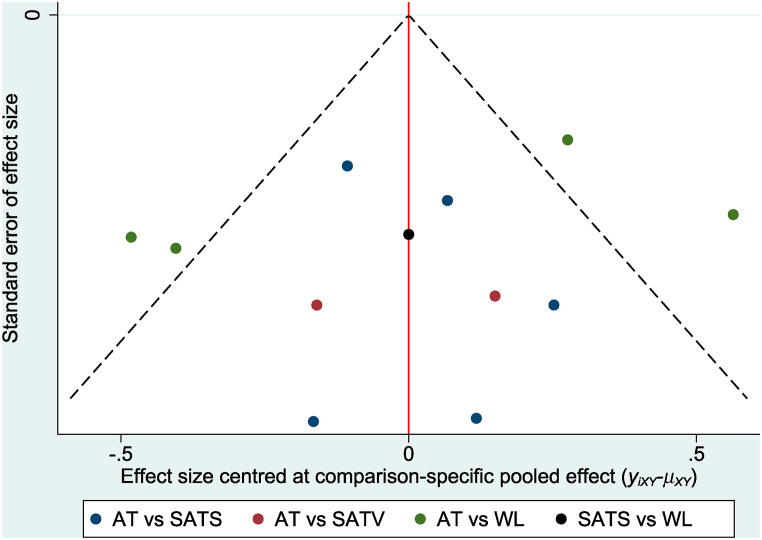
Funnel plot: Pain. AT, acupuncture therapy; SATS, sham acupuncture needling at points different from those in the verum acupuncture group; SATV, sham acupuncture needling at the same acupuncture points as those in the verum acupuncture group; WL, waiting list.

Although the pain intensity of knee osteoarthritis patients significantly improved after treatment with verum acupuncture compared with SATS (SMD 0.41, 95% CI 0.08 to 0.74), the effect of verum acupuncture was not significantly different from that of SATV (SMD 0.00, 95% CI −0.54 to 0.54). In comparison with waiting list, verum acupuncture (SMD -0.67, 95% CI −1.01 to −0.33) and SATV (SMD −0.67, 95% CI −1.31 to −0.04) were significantly better for pain reduction. However, there was no significant difference between SATS and waiting list (SMD −0.26, 95% CI −0.70 to 0.18) and between SATV and SATS (SMD −0.41, 95% CI −1.05 to 0.22) (Table 2 and Fig. 4(A)). The results of NMA and pairwise meta-analysis were mostly consistent; however, the statistically significant difference between waiting list and SATS in pairwise meta-analysis was not observed in NMA (Table 2). The contribution of each direct comparison for mixed and indirect estimates is presented in Supplement 5. The funnel plot was visually symmetric (Fig. 5), and the *P* value was .159 in Egger's test; thus, we assessed that there was low risk of publication bias. Based on the SUCRA for pain reduction, verum acupuncture ranked first at 82.7%, followed by SATV (79.5%), SATS (32.9%), and waiting list (4.9%) (Supplement 6).

**Table 2:** League table for pairwise meta-analysis (right upper part) and network meta-analysis (left lower part) estimates.

**(a) Pain**			
**WL**	**−0.64 (-1.10, -0.17)**	**−0.68 (-1.02, -0.34)**	–
**−0.67 (-1.01, -0.33)**	**AT**	**0.45 (0.29, 0.60)**	0.00 (−0.31, 0.31)
−0.26 (−0.70, 0.18)	**0.41 (0.08, 0.74)**	**SATS**	–
**−0.67 (-1.31, -0.04)**	0.00 (−0.54, 0.54)	−0.41 (−1.05, 0.22)	**SATV**
**(b) Physical function**			
**WL**	**−0.77 (-1.41, -0.12)**	**−0.85 (-1.19, -0.50)**	–
**−0.77 (-1.25, -0.29)**	**AT**	**0.31 (0.08, 0.53)**	−0.08 (−0.53, 0.36)
−0.53 (−1.13, 0.06)	0.24 (−0.21, 0.69)	**SATS**	–
−0.85 (−1.90, 0.19)	−0.08 (−1.01, 0.84)	−0.32 (−1.35, 0.71)	**SATV**

Results are presented as the standardized mean difference (95% confidence interval). The comparison is read from left to right. A standardized mean difference less than zero indicates that treatment on the right is favored in both pairwise and network meta-analyses.

AT, acupuncture therapy; SATS, sham acupuncture needling at points different from those in the verum acupuncture group; SATV, sham acupuncture needling at the same acupuncture points as those in the verum acupuncture group; WL, waiting list.

### 3.5 Data analysis: physical function outcome

Verum acupuncture improved physical function significantly compared with waiting list (SMD -0.77, 95% CI -1.25 to −0.29); however, there was no sdifference between SATV and waiting list (SMD −0.85, 95% CI −1.90 to 0.19) and between SATS and waiting list (SMD −0.53, 95% CI −1.13 to 0.06). In addition, there was no difference in physical function between SATV and verum acupuncture (SMD −0.08, 95% CI −1.01 to 0.84), SATS and verum acupuncture (SMD 0.24, 95% CI −0.21 to 0.69), and SATV and SATS (SMD −0.32, 95% CI −1.35 to 0.71) (Table 2 and Fig. 4(B)). The statistical significance between groups in pairwise meta-analysis was largely lost in NMA (Table 2). The contribution of each direct comparison for mixed and indirect estimates is presented in Supplement 5. It was not appropriate to test for publication bias because fewer than 10 studies were analyzed. Based on the SUCRA plot for function improvement, verum acupuncture ranked first at 75.9%, followed by SATV (75%), SATS (45.9%), and waiting list (3.2%) (Supplement 6). Cluster analysis of the SUCRA for the outcomes of pain and function revealed that verum acupuncture was the best treatment, followed by SATV, SATS, and waiting list (Supplement 7).

### 3.6 Certainty of evidence

The certainty of the direct and indirect evidence between all comparisons for both the pain and physical function was moderate, which was downgraded due to the risk of bias of the included studies. The certainty of evidence for NMA estimates was low to moderate for both outcomes, especially due to imprecision and risk of bias (Table 3).

**Table 3:** Certainty of evidence for the main findings.

(a) Pain				
Comparison		Direct evidence	Indirect evidence	Network meta-analysis
AT	SATV	Moderate Risk of bias (−1)	–	Low Risk of bias (−1) Imprecision (−1)
AT	SATS	Moderate Risk of bias (−1)	Moderate Risk of bias (−1)	Moderate Risk of bias (−1)
AT	WL	Moderate Risk of bias (−1)	Moderate Risk of bias (−1)	Moderate Risk of bias (−1)
SATV	SATS	–	Moderate Risk of bias (−1)	Low Risk of bias (−1) Imprecision (−1)
SATV	WL	–	Moderate Risk of bias (−1)	Moderate Risk of bias (−1)
SATS	WL	Moderate Risk of bias (−1)	Moderate Risk of bias (−1)	Low Risk of bias (−1) Imprecision (−1)
**(b) Physical function**				

Comparison		Direct evidence	Indirect evidence	Network meta-analysis
AT	SATV	Moderate Risk of bias (−1)	–	Low Risk of bias (−1) Imprecision (−1)
AT	SATS	Moderate Risk of bias (−1)	Moderate Risk of bias (−1)	Low Risk of bias (−1) Imprecision (−1)
AT	WL	Moderate Risk of bias (−1)	Moderate Risk of bias (−1)	Moderate Risk of bias (−1)
SATV	SATS	–	Moderate Risk of bias (−1)	Low Risk of bias (−1) Imprecision (−1)
SATV	WL	–	Moderate Risk of bias (−1)	Low Risk of bias (−1) Imprecision (−1)
SATS	WL	Moderate Risk of bias (−1)	Moderate Risk of bias (−1)	Low Risk of bias (−1) Imprecision (−1)

AT, acupuncture therapy; SATS, sham acupuncture needling at points different from those in the verum acupuncture group; SATV, sham acupuncture needling at the same acupuncture points as those in the verum acupuncture group; WL, waiting list.

## 4 Discussion

In previous sham acupuncture-controlled acupuncture trials, although the acupuncture techniques of sham acupuncture are different from those of verum acupuncture (e.g., the use of a sham acupuncture device or minimal acupuncture needling), sham acupuncture needling is sometimes conducted at the same acupuncture points as those in the verum acupuncture. Positive results regarding the specificity of acupuncture point have been reported in functional neuroimaging studies and biological studies [23,24], and simple touch to the acupuncture point has also been shown to induce effects [25]. Therefore, questions have been raised regarding whether sham acupuncture needling at the same acupuncture points as those in the verum acupuncture is a real placebo, considering that both types of acupuncture (sham and verum) induce effects when applied to the acupuncture points [1,3,4]. In a previous study we have demonstrated that trials using sham devices may underrepresent the true effectiveness of acupuncture for knee osteoarthritis in clinical settings [26]. Furthermore, in a previous study of acupuncture for cLBP [5], we confirmed that the effect of sham acupuncture could differ according to needling points. We aimed to investigate if similar results could be obtained for knee osteoarthritis, another major pain condition for which acupuncture has been frequently used and studied. We initially attempted to analyze all pain conditions. However, this did not satisfy the transitivity and clinical similarity assumptions of NMA, so it was performed for a single condition. Although there are several systematic reviews [27,28] of acupuncture for knee osteoarthritis, no study has examined the effects of acupuncture according to the specificity of the sham acupuncture needling points through a NMA. Ten studies were included following a comprehensive search of 10 databases.

Based on the results of NMA for pain outcome, verum acupuncture was significantly superior to SATS but not significantly different from SATV. Additionally, SATV showed a significant difference when compared with waiting list. However, for physical function, no differences between SATS and verum acupuncture, and between SATV and waiting list were found. Based on the SUCRA and clustered ranking plot, which analyzed the priority of the intervention, verum acupuncture ranked first for both pain reduction and function improvement, followed by SATV, SATS, and waiting list. The overall risk of bias of the studies included was either “high” or “some concerns”. In particular, in this study, waiting list groups were included as a comparator, and in the case of studies including waiting list groups, participant blinding was not possible. Therefore, in this case, it was impossible to blind the outcome assessor on the outcome of interest of this study, which is a patient-reported scale. However, our study aimed to examine the effects of acupuncture depending on the needling point of sham acupuncture, and the risk of bias affecting the difference between sham and verum acupunctures, which was the main interest results, was relatively low.

In our study, the differences in effects according to the points needled (sham acupuncture at the same or different acupuncture points compared with those in the verum acupuncture) were not consistent for the pain and function outcomes. Pain perceived by the patient is the most common symptom of knee osteoarthritis, and the pain experience of patients with osteoarthritis directly contributes to the limitation of physical function [29]. Immediate pain reduction in patients after acupuncture has been observed in several studies, and it may lead to improved muscle function and performance [30,31]. Therefore, compared with pain improvement, improvement in physical function may require a relatively longer time. In our study, the results obtained immediately after the end of treatment were used for analysis; thus, the eventual effects of acupuncture on physical function, which may take longer to improve, might not be accurately reflected. However, the included studies did not report the time taken to improve pain and function or their correlation, so this hypothesis needs to be tested through future research.

According to our previous NMA study analyzing the effects of acupuncture depending on the needling points of sham acupuncture for cLBP [5], a significant difference was found between SATS and verum acupuncture, and there was no difference between SATV and verum acupuncture in both pain and function. Additionally, the comparative effectiveness was significant between SATV and SATS for pain and function. NMA results were different for the two conditions (cLBP and knee osteoarthritis) in terms of the physical function outcome. Moreover, the statistical significance of the effect difference between SATV and SATS was different, although the direction of the results was the same in the forest plots of the two conditions. This might be due to the precision of the results due to differences in the total sample size included in the analysis. For cLBP, 4379 populations were analyzed, and for knee osteoarthritis, a relatively small number of 1628 participants were analyzed. Such differences might be attributed to differences in diseases, as well as other factors contributing to the effect of acupuncture, such as patient-doctor interaction, points selected, and needling technique. However, in both cLBP and knee osteoarthritis, the outcome of sham acupuncture on pain was different from that of verum acupuncture depending on whether sham acupuncture was performed at the same acupuncture points as those in verum acupuncture. Therefore, the efficacy of acupuncture might have been underestimated in previous trials due to inaccurate point selection in sham acupuncture for the two representative musculoskeletal conditions for which pain is the main symptom.

Sham acupuncture sometimes incorporates sham acupuncture devices (base units), such as the Park sham acupuncture device [32], and these studies must use the base units in the verum acupuncture arm to blind the participants. In our previous NMA, verum acupuncture in sham acupuncture-controlled acupuncture trials without the base units was more effective than that in sham acupuncture-controlled acupuncture trials using the base units for improving pain and function [26]. Among the studies included in this NMA, the Park sham device was used as a sham acupuncture device in only one article [16] in the SATV group, and the sham acupuncture device was not used in the SATS group. In addition, in this NMA, which included only 10 studies, the additional effect of using a sham acupuncture device could not be analyzed because it increased the nodes of the network map and affected the precision of the study results.

A limitation of this study is that there were only 10 included studies, which might affect the precision of the analysis performed on the 4-node network map. Therefore, it may be difficult to conduct additional NMA studies of other effect modifiers.

Nevertheless, this was the first study to analyze the outcomes of acupuncture according to the points needled in sham acupuncture for knee osteoarthritis, and the results were similar to those of a comparable analysis for cLBP [5]. In addition, we searched not only an English database but also Chinese, Japanese, and Korean local databases to include relevant literature as comprehensively as possible. Furthermore, our approach was methodologically rigorous and comprehensive considering that we assessed the risk of bias and the certainty of evidence and produced contribution plots and ranking plots for effect estimates. It is possible to determine whether there is an actual difference in effects on pain outcome between verum acupuncture, SATV, and SATS through direct comparative clinical trials, which would confirm the accuracy of our NMA results. However, as questions continue to arise as to whether sham acupuncture can be used as a real placebo, it may be more helpful to confirm whether there is a difference in the outcome of acupuncture based on the specificity of needling points of sham acupuncture in other pain conditions. Furthermore, a NMA may be conducted to determine whether consistent results are obtained in non-pain conditions. This would help elucidate mechanisms related to the physiological activity of sham acupuncture, which is currently misused as placebo control to assess the efficacy of acupuncture.

## 5 Conclusion

For patients with knee osteoarthritis, the pain reduction effect of acupuncture may differ depending on the sham acupuncture needling points, and any stimulation at the same acupuncture points as those in the verum acupuncture arm may not be a true placebo for evaluating the efficacy of acupuncture. The control group should be established according to the specific aim of the study design and treatment mechanism.

## Funding statement and role of the funding sources

This work was supported by the 10.13039/501100003718Korea Institute of Oriental Medicine (KSN2311021, KSN1823211, KSN2121211 and KSN23314112). Funding sources had no involvement in study design; in the collection, analysis and interpretation of data; in the writing of the report; or in the decision to submit the article for publication.

## Data availability statement

The authors confirm that the data supporting the findings of this study are available within the article and its supplementary materials.

## CRediT authorship contribution statement

**Boram Lee:** Writing – original draft, Methodology, Formal analysis, Conceptualization. **Chan-Young Kwon:** Writing – review & editing, Methodology, Conceptualization. **Hye Won Lee:** Writing – review & editing. **Arya Nielsen:** Writing – review & editing. **L Susan Wieland:** Writing – review & editing. **Tae-Hun Kim:** Writing – review & editing. **Stephen Birch:** Writing – review & editing. **Terje Alraek:** Writing – review & editing. **Myeong Soo Lee:** Writing – review & editing, Visualization, Supervision, Methodology, Conceptualization.

## Declaration of competing interest

The authors declare that they have no known competing financial interests or personal relationships that could have appeared to influence the work reported in this paper.
