# Supplementary material for: The effect of sham acupuncture can differ depending on the points needled in knee osteoarthritis: A systematic review and network meta-analysis
Source: Heliyon. 2024 Feb 7;10(4):e25650. doi: 10.1016/j.heliyon.2024.e25650 (PMC10877264; doi:10.1016/j.heliyon.2024.e25650)
Supplement: Multimedia component 1 [file mmc1.docx]

**Supplementary Online Contents**

**Supplement 1. Search strategy used in each database**

**Supplement 2. Excluded studies after full text review**

**Supplement 3. Details of verum acupuncture method**

**Supplement 4. Results of testing inconsistency at the local level through the node splitting method**

**Supplement 5. Contribution plots**

**Supplement 6. SUCRA plots**

**Supplement 7. Clustered ranking plot based on cluster analysis of SUCRA values for two different outcomes: pain and physical function**

**Supplement 1. Search strategy used in each database**

**Medline via PubMed**

|  | Searches | Results |
| --- | --- | --- |
| #1 | "Osteoarthritis, Knee"[MH] OR ((osteoarthr*[TIAB] OR arthrosis[TIAB] OR "degenerative arthr*"[TIAB]) AND (Knee[MH] OR knee[TIAB] OR "Knee Joint"[MH])) OR KOA[TIAB] OR gonarthr*[TIAB] OR "knee pain"[TIAB] | 52,972 |
| #2 | Acupuncture[MH] OR “Acupuncture Therapy”[MH] OR “Acupuncture Points”[MH] OR acupunct*[TIAB] OR acupoint*[TIAB] OR “Dry Needling”[MH] OR “dry needling”[TIAB] OR "filiform needle"[TIAB] | 37,564 |
| #3 | “Randomized Controlled Trial”[PT] OR “Controlled Clinical Trial”[PT] OR randomized[TIAB] OR placebo[TIAB] OR “Clinical Trials as Topic”[Mesh: noexp] OR randomly[TIAB] OR trial[TI] | 1,594,514 |
| #4 | animals[MH] NOT humans[MH] | 5,086,137 |
| #5 | (#1 AND #2 AND #3) NOT #4 | **469** |

**EMBASE via Elsevier**

|  | Searches | Results |
| --- | --- | --- |
| #1 | 'knee osteoarthritis'/exp 'knee osteoarthritis':ab,ti OR 'knee arthritis'/exp OR 'knee arthritis':ab,ti OR ((osteoarthr*:ab,ti OR arthrosis:ab,ti OR 'degenerative arthr*':ab,ti) AND (knee/exp OR knee:ab,ti)) OR KOA:ab,ti OR gonarthr*:ab,ti OR 'knee pain'/exp OR 'knee pain':ab,ti | 82,776 |
| #2 | acupuncture/exp OR acupuncture*:ab,ti OR ‘acupuncture point’/exp OR ‘body meridian’/exp OR ‘body meridian’:ab,ti OR acupoint*:ab,ti OR 'dry needling'/exp OR 'dry needling':ab,ti OR ‘filiform needle’:ab,ti | 61,133 |
| #3 | 'crossover procedure':de OR 'double-blind procedure':de OR 'randomized controlled trial':de OR 'single-blind procedure':de OR (random* OR factorial* OR crossover* OR cross NEXT/1 over* OR placebo* OR doubl* NEAR/1 blind* OR singl* NEAR/1 blind* OR assign* OR allocat* OR volunteer*):de,ab,ti | 3,073,826 |
| #4 | #1 AND #2 AND #3 | **819** |

**CENTRAL**

|  | Searches | Results |
| --- | --- | --- |
| #1 | MeSH descriptor: [Osteoarthritis, Knee] explode all trees | 5,304 |
| #2 | (((osteoarthr* OR arthrosis OR "degenerative arthr*") AND Knee) OR KOA OR Gonarthr* OR "Knee pain"):ti,ab,kw | 16,765 |
| #3 | #1 OR #2 | 16,765 |
| #4 | MeSH descriptor: [Acupuncture] explode all trees | 167 |
| #5 | MeSH descriptor: [Acupuncture Therapy] explode all trees | 5,394 |
| #6 | MeSH descriptor: [Acupuncture Points] explode all trees | 2,292 |
| #7 | MeSH descriptor: [Dry Needling] explode all trees | 110 |
| #8 | (acupunct* OR acupoint* OR “dry needling” OR "filiform needle"):ti,ab,kw | 20,235 |
| #9 | #4 OR #5 OR #6 OR #7 OR #8 | 20,496 |
| #10 | (#3 AND #9) in Trials | **746** |

**AMED via EBSCO**

|  | Searches | Results |
| --- | --- | --- |
| #1 | "Osteoarthritis, Knee"[SU] OR KOA[TX] OR gonarthr*[TX] OR "knee pain"[TX] | 2,042 |
| #2 | (osteoarthr*[TX] OR arthrosis[TX] OR "degenerative arthr*"[TX]) AND (Knee[SU] OR knee[TX] OR "Knee Joint"[SU]) | 2,636 |
| #3 | Acupuncture[SU] OR “Acupuncture Therapy”[SU] OR “Acupuncture Points”[SU] OR acupunct*[TX] OR acupoint*[TX] OR “Dry Needling”[SU] OR “dry needling”[TX] OR "filiform needle"[TX] | 12,353 |
| #4 | (#1 OR #2) AND #3 | **172** |

**OASIS**

|  | Searches | Results |
| --- | --- | --- |
| #1 | (무릎\|슬관절\|슬통\|골관절염\|퇴행성관절염) (침) | **3** |

**KISS**

|  | Searches | Results |
| --- | --- | --- |
| #1 | 제목=(무릎\|슬관절\|슬통\|골관절염\|퇴행성관절염) AND 제목=(침) | **102** |

**KMbase**

|  | Searches | Results |
| --- | --- | --- |
| #1 | (((([TITLE=무릎] OR [TITLE=슬관절]) OR [TITLE=슬통]) OR [TITLE=골관절염]) OR [TITLE=퇴행성관절염]) | 2,767 |
| #2 | [TITLE=침] | 4,574 |
| #3 | #1 AND #2 | **47** |

**ScienceON**

|  | Searches | Results |
| --- | --- | --- |
| #1 | 논문명=(무릎\|슬관절\|슬통\|골관절염\|퇴행성관절염) (침) | **96** |

**CNKI**

|  | Searches | Results |
| --- | --- | --- |
| #1 | (SU=’膝关节炎’+'膝骨关节炎’+'膝骨性关节炎’+'膝关节骨关节炎’+'膝关节骨性关节炎’+'膝通’) and (SU=’针’) | **2,263** |

**CiNii**

|  | Searches | Results |
| --- | --- | --- |
| #1 | (膝関節炎 OR 膝骨関節炎 OR 膝変形性関節症 OR 膝の変形性関節症 OR 膝の痛み) AND 鍼 | **33** |

**Supplement 2. Excluded studies after full text review**

**1) Nonrandomized controlled trials (n = 25)**

1. 江育清, 莫倩云, and 杨顺益, *退行性膝关节炎针灸治疗研究进展.* 中国民间疗法, 2002(12): p. 57-58.

2. 景传生, *中西医结合治疗骨性膝关节炎.* 深圳中西医结合杂志, 2000(03): p. 125-129.

3. 唐华伟, *温阳通经法治疗老年性膝关节炎78例临床观察.* 中医正骨, 2005(09): p. 69.

4. 杜昌华, *耳穴埋针治疗骨质增生性膝关节炎30例.* 浙江中医杂志, 1999(04): p. 166.

5. 廖仲围, *针刺治疗膝关节炎136例.* 中国民间疗法, 2001(10): p. 14.

6. 林雷 and 张华, *中西医结合治疗退行性膝关节炎.* 少年体育训练, 2008(01): p. 56-57.

7. 林耀庚, *针刺大巨穴治疗增生性膝关节炎40例.* 天津中医, 1999(05): p. 29-30.

8. 徐江文, *中西医结合治疗骨性膝关节炎.* 实用中西医结合临床, 2004(05): p. 56-57.

9. 王文欣 and 黄琳娜, *膝四针为主治疗增生性膝关节炎38例.* 中国针灸, 2000(11): p. 16.

10. 王淑琴, 姜丽, and 赵淑杰, *针刺为主治疗骨性膝关节炎49例.* 吉林医学, 2005(06): p. 610.

11. 宁玲 and 范庆花, *针灸治疗增生性膝关节炎365例.* 中国民间疗法, 2002(07): p. 10-11.

12. 曾焕芝, *中西医结合治疗马属动物变形性膝关节炎.* 当代畜牧, 2010(09): p. 21-22.

13. 曾焕芝, *中西结合治疗马属动物变形性膝关节炎.* 中兽医医药杂志, 2011. **30**(01): p. 53-54.

14. 彭易雨, *针刺膝眼穴为主治疗膝关节炎120例.* 实用中医药杂志, 2002(01): p. 30-31.

15. 刘长信. *退行性膝关节炎的中医综合疗法*. in *2012中国康复医学会实用康复论坛暨运动康复新理念培训班*. 2012. 中国河南安阳.

16. 劳力行. *针刺治疗膝关节炎疗效的临床试验*. in *第三届国际中医药工程学术会议*. 2006. 中国上海.

17. 张建平, *针灸温通法治疗退行性膝关节炎:针具不同疗效有差异.* 中国临床康复, 2002(16): p. 2464.

18. 张丁丁, *针灸疗法可减轻膝关节炎患者疼痛并改善关节功能.* 国外医学情报, 2005(04): p. 16-17.

19. 张洪军 and 郭明芳, *针刺为主治疗增生性膝关节炎200例.* 上海针灸杂志, 2002(03): p. 45.

20. Boylan, M., *Acupuncture with Voltaren is superior than Voltaren alone in knee osteoarthritis.* J Aust Tradit Med Soc, 2005. **11**(2).

21. Fargas-Babjak, A.M., B. Pomeranz, and P.J. Rooney, *Acupuncture-like stimulation with codetron for rehabilitation of patients with chronic pain syndrome and osteoarthritis.* Acupuncture & electro-therapeutics research, 1992. **17**(2): p. 95‐105.

22. Jiang, A., et al., *Clinical effect of acupuncture treatment in 109 cases of knee osteoarthritis.* Journal of Traditional Chinese Medicine, 2001. **21**(4): p. 282-5.

23. Liu, T. and C. Liu, *Acupuncture for treating osteoarthritis of the knee and the hip.* Arthritis and Rheumatism, 2006. **54**(11): p. 3375-3377.

24. Tebbutt, E., *Osteoarthritis of the knee in an elderly patient, treated with acupuncture.* J Acupunct Assoc Chart Physiotherapists, 2004. **2004**(1): p. 51-8.

25. Zhou, S.F. and C.C. Xue, *Acupuncture as an adjunct to exercise-based physiotherapy does not improve the pain of knee osteoarthritis.* Australian journal of acupuncture and chinese medicine, 2008. **3**(1): p. 53‐55.

**2) Not only for patients with knee osteoarthritis (n = 4)**

1. Christensen, B.V., et al., *Acupuncture treatment of severe knee osteoarthrosis. A long-term study.* Acta anaesthesiologica Scandinavica, 1992. **36**(6): p. 519‐525.

2. Christensen, B.V., et al., *Acupuncture treatment of knee arthrosis. A long-term study.* Ugeskrift for laeger, 1993. **155**(49): p. 4007‐4011.

3. Huang, W., et al., *Acupuncture for pain and sleep in knee osteoarthritis.* Journal of the American Geriatrics Society, 2010. **58**(6): p. 1218‐1220.

4. Molsberger, A., et al., *[Acupuncture treatment for the relief of gonarthrosis pain-a controlled clinical trial.].* Schmerz, 1994. **8**(1): p. 37-42.

**3) Not about only manual acupuncture (n = 19)**

1. 徐杰, et al., *中西医结合治疗增生性膝关节炎34例.* 医学理论与实践, 2004(05): p. 549.

2. 鲍圣涌, 张少君, and 陈竞芬, *中西医结合治疗增生性膝关节炎的临床观察.* 湖北中医药大学学报, 2012. **14**(02): p. 52-53.

3. Berman, B.M., et al., *Effectiveness of acupuncture as adjunctive therapy in osteoarthritis of the knee: a randomized, controlled trial.* Annals of internal medicine, 2004. **141**(12): p. 901‐910.

4. Berman, B.M., et al., *A randomized trial of acupuncture as an adjunctive therapy in osteoarthritis of the knee.* Rheumatology (Oxford, England), 1999. **38**(4): p. 346‐354.

5. Jubb, R.W., et al., *A blinded randomised trial of acupuncture (manual and electroacupuncture) compared with a non-penetrating sham for the symptoms of osteoarthritis of the knee.* Acupuncture in medicine, 2008. **26**(2): p. 69‐78.

6, Mavrommatis, C.I., et al., *Acupuncture as an adjunctive therapy to pharmacological treatment in patients with chronic pain due to osteoarthritis of the knee: a 3-armed, randomized, placebo-controlled trial.* Pain, 2012. **153**(8): p. 1720‐1726.

7. Suarez-Almazor, M.E., et al., *A randomized controlled trial of acupuncture for osteoarthritis of the knee: Effects of patient-provider communication.* Arthritis Care and Research, 2010. **62**(9): p. 1229-1236.

8. Vas, J., C. Mendez, and E. Perea-Milla, *Acupuncture versus Streitberger needle in knee osteoarthritis -- an RCT.* Acupuncture medicine, 2006. **24**(Suppl): p. S15‐S24.

9. Vas, J., et al., *Acupuncture as a complementary therapy to the pharmacological treatment of osteoarthritis of the knee: randomised controlled trial.* BMJ (Clinical research ed.), 2004. **329**(7476): p. 1216.

10. White, A., et al., *Western medical acupuncture in a group setting for knee osteoarthritis: results of a pilot randomised controlled trial.* Pilot Feasibility Stud, 2016. **2**: p. 10.

11. Farazdaghi, M., et al., *Dry needling trigger points around knee and hip joints improves function in patients with mild to moderate knee osteoarthritis.* Journal of bodywork and movement therapies, 2021. **27**: p. 597‐604.

12. Romero, E.A.S., et al., *Is a Combination of Exercise and Dry Needling Effective for Knee OA?* Pain medicine (Malden, Mass.), 2020. **21**(2): p. 349‐363.

13. Sánchez-Romero, E.A., et al., *Effects of dry needling in an exercise program for older adults with knee osteoarthritis.* Medicine (United States), 2018. **97**(26).

14. Vervullens, S., et al., *The effect of one dry needling session on pain, central pain processing, muscle co-contraction and gait characteristics in patients with knee osteoarthritis: a randomized controlled trial.* Scand J Pain, 2022. **22**(2): p. 396-409.

15. Chen, L.X., et al., *Integrating acupuncture with exercise-based physical therapy for knee osteoarthritis: a randomized controlled trial.* Journal of clinical rheumatology, 2013. **19**(6): p. 308‐316.

16. Foster, N.E., et al., *Acupuncture as an adjunct to exercise based physiotherapy for osteoarthritis of the knee: randomised controlled trial.* BMJ (Clinical research ed.), 2007. **335**(7617): p. 436.

17. Lev-Ari, S., et al., *Delayed effect of acupuncture treatment in OA of the knee: a blinded, randomized, controlled trial.* Evidence-based complementary and alternative medicine, 2011. **2011**.

18. Penagos-Martinez, A.A., B.E. Patino-Palma, and O.L. Rodriguez-Puerto, *Acupuncture and therapeutic exercise in women over 50 with osteoarthritis of the knee. Randomized clinical trial.* Revista Internacional de Acupuntura, 2021. **15**(1): p. 15‐23.

19. Scharf, H.P., et al., *Acupuncture and knee osteoarthritis: a three-armed randomized trial.* Annals of internal medicine, 2006. **145**(1): p. 12‐20.

**4) Comparison between verum acupuncture and active control (n = 5)**

1. 涂小华, *毫针傍刺治疗退行性膝关节炎.* 现代医药卫生, 2002(09): p. 801.

2. 李保军 and 杨巧凤, *针刺为主治疗增生性膝关节炎120例.* 上海针灸杂志, 2002(03): p. 31.

3. 徐菁 and 张大同, *阿是穴避痛埋针治疗老年退行性膝关节炎的疗效观察.* 中国中医药科技, 2016. **23**(03): p. 355-356.

4. 肖建墙. *关节腔内注射玻璃酸钠联合针灸治疗骨性膝关节炎的效果评价*. in *国际数字医学会数字中医药分会成立大会暨首届数字中医药学术交流会*. 2016. 中国广东珠海.

5. Zhang, J.-G., *Clinical effect observation of knee joint degenerative osteoarthropathy treated with acupuncture.* Journal of clinical acupuncture and moxibustion, 2003. **19**(11): p. 21.

**5) No data on outcomes of interest (n = 3)**

1. Itoh, K., et al., *Trigger point acupuncture for treatment of knee osteoarthritis--a preliminary RCT for a pragmatic trial.* Acupuncture in medicine, 2008. **26**(1): p. 17‐26.

2. Karner, M., et al., *Objectifying specific and nonspecific effects of acupuncture: a double-blinded randomised trial in osteoarthritis of the knee.* Evidence-based complementary and alternative medicine, 2013. **2013**.

3. Spaeth, R.B., et al., *A longitudinal study of the reliability of acupuncture deqi sensations in knee osteoarthritis.* Evidence-based complementary and alternative medicine, 2013. **2013**.

**6) Duplicate data (n = 6)**

1. 古屋, 英. and 美. 直本, *Acupuncture for osteoarthritis of the knee.* 医道の日本 = The Japanese journal of acupuncture & manual therapies : 東洋医学・鍼灸マッサージの専門誌, 2012. **71**(10): p. 71-73.

2. Brinkhaus, B., et al., *Physician and treatment characteristics in a randomised multicentre trial of acupuncture in patients with osteoarthritis of the knee.* Complementary therapies in medicine, 2007. **15**(3): p. 180‐189.

3. Brinkhaus, B., et al., *Efficacy of acupuncture in patients with osteoarthritis of the knee. A randmized controlled trial.* Gynakologische Praxis, 2006. **30**(3): p. 539‐549.

4. Foster, N.E., et al., *The relationship between patient and practitioner expectations and preferences and clinical outcomes in a trial of exercise and acupuncture for knee osteoarthritis.* European journal of pain (London, England), 2010. **14**(4): p. 402‐409.

5. Manheimer, E., et al., *Acupuncture for knee osteoarthritis - a randomised trial using a novel sham.* Acupuncture in medicine, 2006. **24**: p. 7‐14.

6. Tu, J. F., et al., *Effect of acupuncture on knee injury and osteoarthritis outcome score in patients with knee osteoarthritis*. Zhongguo zhen jiu [Chinese acupuncture & moxibustion], 2021. **41**(1): p. 27‐30.

**Supplement 3. Details of verum acupuncture method**

| **Study ID (First author, year)** | **Acupuncture rationale** | **Acupuncture protocol** | **Acupuncture points** | **Depth of insertion** | **Needle retention time** | **Needle size** | **De-qi** | **Treatment frequency** | **Number of treatment session** |
| --- | --- | --- | --- | --- | --- | --- | --- | --- | --- |
| Hinman 2014 | TCM | Semi-standardized | SP9, SP10, ST34, ST35, ST36, LR7, LR8, LR9, KI10, BL39, BL40, BL57, GB34, GB35, GB36, local extra points in the hamstring muscles, ST40, LR3, SP6, GB41, BL60, BL21, BL22, BL23, GB30, GB31, ear knee point, DU20, LI11, GV14, and BL11 *Acupuncturists were permitted to treat both knees. | Not recorded | 20 min | 0.25 × 40 mm | Not recorded | once or twice a week | 8~12 |
| Lam 2021 | TCM | Individualized | Acupuncture points (1–2 cm away from the point of tenderness, spasm or pain) along the meridian sinews near the knee *affected side | 10-20 mm | 30 min | 0.30 x 40 mm | Not recorded | three times a week (first 2 weeks), twice a week (next 2 weeks) | 10 |
| Lin 2018 | TCM | Semi-standardized | ST34, ST35, ST36, EX-LE2, EX-LE5, GB33, GB34, SP9, SP10, LR8, GB31, GB36, GB39, GB41, ST40, ST41, LR3, BL60, SP6, KI3, and LI4 *affected side | 10-30 mm | 20 min | 0.30 × 25 mm 0.30 × 40 mm | Achieving | 3 times a week | 24 |
| Min 2006 | TCM | Individualized | -Pain at upper and dorsal to the medial condyle of the tibia: HT8, SP2, LR1, and SP1 -Pain at the medial end of the popliteal crease, dorsal to the medial condyle of the tibia: KI10, LR8, LU8, and LR4 -Pain at ventral and distal to the head of the fibula: BL66, GB43, LI1, and GB44 -Pain in the middle of the popiliteal fibula crease: LI1, BL67, ST36, and BL40 -Pain at the lower edge of the patella, lateral to the patella ligament: SI5, LI5, GB41, and ST43 -Pain in the medial part of the popliteal fossa between the tendons of the semi-tendinosus and semi-membranosus muscles: LU8, KI7, SP3, and ST41 *opposite side | 1-5 mm | 20 min | 0.25 × 40 mm | Achieving | twice a week | 8 |
| Takeda 1994 | TCM | Standardized | SP9, ST35, GB34, Extra 31, and Extra 32 | Not recorded | 30 min | 0.23 x 30 mm | Achieving | three times a week | 9 |
| Tu 2021 | TCM | Semi-standardized | ST35, EX-LE5, LR8, GB33, Ah-shi point *3 additional points among ST32, ST34, SP10, EX-LE2, ST36, ST40, LR3, LR7, LR9, SP6, KI3, SP4, BL39, BL57, BL40, KI10, GB31, GB34, GB36, GB39, BL60, and GB41 *affected side | ≥ 10 mm | 30 min | 0.25 x 25-40 mm | Achieving | three times a week | 24 |
| Williamson 2007 | TCM | Semi-standardized | SP10, ST35, EX-LE5, ST36, SP9, GB34, and LR3 | Not recorded | 20 min | 1 inch, 0.25 gauge | Achieving | once a week | 6 |
| Witt 2005 | TCM | Semi-standardized | -at least 6 local points among ST34, ST35, ST36, SP9, SP10, BL40, KI10, GB33, GB34, LR8, EX-LE2, and EX-LE5 -at least 2 distant points among SP4, SP5, SP6, ST6, BL20, BL57, BL58, BL60, BL62, and KI3 | Not recorded | 30 min | 20-40 mm length | Achieving | twice a week (first 4 weeks), once a week (next 4 weeks) | 12 |
| Witt 2006 | TCM | Individualized | Not recorded | Not recorded | Not recorded | Not recorded | Not recorded | Not recorded | 15 |
| Yu 2021 | TCM | Standardized | LI11, HT3, TE10 or GB34, SP9, and EX-LE2 *bilateral | 15-30 mm | 20 min | 0.25 × 40 mm | Not recorded | once | 1 |

Abbreviation. TCM, traditional Chinese medicine.

Supplement 4. Results of testing inconsistency at the local level through the node splitting method

1. Pain

| Side | Direct | | Indirect | | Difference | | p-value |
| --- | --- | --- | --- | --- | --- | --- | --- |
|  | Coefficient | SE | Coefficient | SE | Coefficient | SE |  |
| SATS WL | 0.678812 | 0.3380768 | 0.0027582 | 0.267874 | 0.6760538 | 0.4314766 | 0.117 |

1. Physical function

| Side | Direct | | Indirect | | Difference | | p-value |
| --- | --- | --- | --- | --- | --- | --- | --- |
|  | Coefficient | SE | Coefficient | SE | Coefficient | SE |  |
| SATS WL | 0.8492684 | 0.4565962 | 0.2642261 | 0.4233884 | 0.5850423 | 0.6226187 | 0.347 |

Abbreviations. SATS, sham acupuncture needling at points different from those in the verum acupuncture group; WL, waiting list.

**Supplement 5. Contribution plots**

1. Pain

1. Physical function

AT, verum acupuncture therapy; SATS, sham acupuncture needling at points different from those in the verum acupuncture group; SATV, sham acupuncture needling at the same acupuncture points as those in the verum acupuncture group; WL, waiting list.

**Supplement 6. SUCRA plots**

1. Pain

1. Physical function

AT, verum acupuncture therapy; SATS, sham acupuncture needling at points different from those in the verum acupuncture group; SATV, sham acupuncture needling at the same acupuncture points as those in the verum acupuncture group; WL, waiting list.

**Supplement 7. Clustered ranking plot based on cluster analysis of SUCRA values for two different outcomes: pain and physical function**

A, verum acupuncture therapy; B, sham acupuncture needling at points different from those in the verum acupuncture group; C, sham acupuncture needling at the same acupuncture points as those in the verum acupuncture group; D, waiting list.
